# Hyponatremia and Renal Venous Congestion in Heart Failure Patients

**DOI:** 10.1155/2021/6499346

**Published:** 2021-08-12

**Authors:** Alexandru Caraba, Stela Iurciuc, Andreea Munteanu, Mircea Iurciuc

**Affiliations:** ^1^Department of Internal Medicine, University of Medicine and Pharmacy “Victor Babeș” Timișoara, Romania; ^2^Department of Cardiology, University of Medicine and Pharmacy “Victor Babeș” Timișoara, Romania

## Abstract

**Objective:**

The interrelationship between the heart and kidneys has a great importance in the homeostasis of the cardiovascular system. Heart failure patients present intrarenal arterial hypoperfusion and intrarenal venous congestion due to reduced left ventricle ejection fraction, which triggers numerous neurohormonal factors. The aim of this study was to investigate intrarenal vascularization (arterial and venous), as well as the links between it and systemic congestion and, on the other side, with the mortality in patients with heart failure. *Material and Methods*. This cross-sectional study was performed on a group of 44 patients with heart failure in different stages of evolution and 44 healthy subjects, matched for age and gender, as controls. Serum natremia, NT-proBNP, and creatinine analyses were performed in all patients and controls. Renal and cardiac ultrasonography was done in all patients and controls, recording intrarenal arterial resistive index (RRI), intrarenal venous flow (IRVF) pattern, renal venous stasis index (RVSI), and left ventricular ejection fraction (LVEF). Data are recorded and presented as mean ± standard deviation. Statistical analyses were performed using the Student *t*-test, ANOVA test, and the Pearson correlation. Differences were considered statistically significant at the value of *p* < 0.05.

**Results:**

Hyponatremia was identified in 47.72% of the HF patients. This study revealed correlations between serum natremia and LVEF, NT-proBNP, serum creatinine, interlobar venous RVSI (*p* < 0.00001), and interlobar artery RRI (*p* ≤ 0.002). Hyponatremia and renal venous congestion represent negative prognostic factors in HF patients.

**Conclusion:**

In HF patients, hyponatremia was correlated with cardiac dysfunction and intrarenal venous congestion. Hyponatremia and renal venous congestion represented negative prognostic factors in HF patients.

## 1. Introduction

Heart failure (HF) is the result of cardiovascular disease evolution, having a poor prognosis with repeated hospitalization, increased morbidity and mortality, and high medical costs [1]. HF incidence is about 1 to 9 cases/1000 person-years, depending on the studied groups of population and, on the other hand, on the diagnostic criteria used. It is estimated that about 64.3 million people are recorded as having HF [2].

Irrespective of its etiology, cardiac dysfunction generates a reduction in arterial perfusion and passive congestion in several organs, causing other clinical manifestations in addition to those caused by heart disease. Some of these manifestations are associated with an unfavorable prognosis and reduced survival of HF patients [3].

The interrelationship between the heart and kidneys has a great importance in the homeostasis of the cardiovascular system [4]. Interrelation between cardiac and renal dysfunctions is known as cardiorenal syndrome [5].

Decreased cardiac output and systemic hypoperfusion generate neurohormonal activation (sympathetic nervous system and the renin-angiotensin-aldosterone system) in order to preserve the systemic perfusion pressure. But in HF patients, these systems act in a maladaptive way, generating excessive retention of sodium and water, perpetuating systemic congestion. On the other side, angiotensin II inhibits the sensation of thirst, leading to increased free water intake and exacerbation of hyponatremia [6, 7]. Hyponatremia is common among patients with HF, having a negative prognosis on survival and readmissions of these patients [8]. Hyponatremia, more often dilutional, is found in about 20-27% of HF patients upon admission. It represents a sign of systemic congestion in HF patients [9].

Volume overload which characterizes HF causes the secretion by the myocardium an amino-terminal pro-B-type natriuretic peptide (NT-proBNP), as a response to myocardial stretch. The levels of NT-proBNP are elevated in HF patients, providing a useful biomarker of cardiac dysfunction [10].

The kidney vascularization in HF is characterized by arterial hypoperfusion and venous congestion. Intrarenal arterial vascularization is assessed by means of interlobar artery ultrasonography and intrarenal resistive index (RRI) providing information about renal function and prognosis in both renal and cardiac diseases [11, 12]. But the studies performed in recent years on patients with HF have shown that the renal function impairment is not only determined by intrarenal arterial hypoperfusion, evaluated by means of RRI, but much more by intrarenal venous congestion [13, 14].

Intrarenal venous flow (IRVF) is influenced by the structure of the surrounding kidney parenchymal histology and the pressure in the inferior caval vein. Systemic congestion and subsequent renal congestion, which characterize HF have influence on IRVF profile. Studying IRVF by means of intrarenal Doppler ultrasonography on interlobar veins, in HF patients, were described by several patterns: continuous, discontinuous biphasic, or monophasic, correlated with right atrial pressure and having prognostic value [15].

The aim of this study was to investigate intrarenal vascularization (arterial and venous), as well as the links between it and systemic congestion and, on the other side, with the mortality in patients with heart failure.

## 2. Material and Methods

### 2.1. Patients

The present study is a cross-sectional one, which was performed in the Department of Internal Medicine, Timișoara, Romania, between January 2018 and May 2021 on a group of 44 patients with HF in different stages of evolution and 44 healthy subjects, matched for age and gender, as controls. All patients fulfilled the classification criteria of HF [16, 17].

Exclusion criteria were represented by age under 18 years, patients' refusal to participate in this study, acute decompensate HF, HF with preserved ejection fraction, primary or secondary pulmonary hypertension, secondary cardiomyopathies, previous acute or chronic kidney diseases, pregnant or breastfeeding women, endocrine diseases, current smokers, and inadequate images of intrarenal vascularization. Control subjects were identified among healthy relatives of patients with HF, without any cardiovascular disease. Informed consent was obtained from all the patients and controls. The study was approved by the Ethics Committee of Railway Clinical Hospital Timișoara, Romania, with registration number 23/January 2018. This study respects the Declaration of Helsinki.

### 2.2. Methods

Serum natremia, NT-proBNP, and creatinine analyses were performed in all patients and controls.

Serum natremia analysis was done using ion selective electrode (ISE) method, normal values being between 136 and 145 mMol/l.

The values of NT-proBNP were assessed by immunochemistry with electrochemiluminescence detection (ECLIA); the value < 300 pg/ml has a negative predictive value of 99% for the exclusion of congestive HF in all patients.

Serum creatinine analysis was done using colorimetric enzymatic Jaffe method (normal values being between 0.6 and 1.2 mg/dl), and glomerular rate filtration (eGRF) was estimated by MDRD formula (http://www.mdrd.com) (normal values over 90 ml/min/1.73 m^2^).

Renal ultrasonography was performed in all the patients and controls, using Siemens ACUSON A2000 or Samsung HS50 with a 3.5 MHz convex transducer. This investigation was performed under fasting conditions for about 6 hours. Intrarenal arterial vascularization was measured on interlobar renal arteries, determining the RRI value at the upper, middle, and lower portions of the kidney in a supine position and was averaged for each kidney. The mean RRI value of both kidneys was recorded. Under normal conditions, the RRI value is less than 0.70 [18]. Intrarenal venous vascularization was done on the interlobar veins, using the same equipment, in the same conditions. IRVF pattern was recorded. Normally, the IRVF pattern is continuous. Increased systemic and intrarenal congestion determines the discontinuous pattern of IRVF, in the form of pulsatile, biphasic, and monophasic. Then, the renal venous stasis index (RVSI) analysis was performed at the upper, middle, and lower portions of the kidney and calculated using the following formula: (cardiac cycle time [msec] − venous flow time [msec])/cardiac cycle time [msec] [7]. The mean value of RVSI of both kidneys was recorded.

Transthoracic cardiac ultrasonography was done using Samsung HS50 with a 2.5 MHz cardiac transducer, based on current recommendations [19]. LVEF was determined in all patients and controls, using the biplane Simpson method. Based on the guideline from the British Society of Echocardiography, LVEF was considered normal (LVEF ≥ 55%), borderline low LVEF (LVEF 50-54%), impaired LVEF (LVEF 36-49%), and severely impaired LVEF (LVEF ≤ 35%) [20].

### 2.3. Statistical Analysis

Data are recorded and presented as mean ± standard deviation. Statistical analyses were performed using the Student *t*-test, ANOVA test, and the Pearson correlation. Differences were considered statistically significant at the value of *p* < 0.05.

## 3. Results

Table 1 presents the demographic data of the patients and controls.

Based on New York Heart Association (NYHA) classification, the studied patients were classified as class I (10 patients), class II (12 patients), class III (11 patients), and class IV (11 patients).

The group of HF patients presented low values of left ventricular ejection fraction (LVEF), serum natremia, and eGFR, having statistical significance (*p* < 0.0001). The same patients presented high values of NT-proBNP and serum creatinine. All these differences were statistically significant (*p* < 0.0001) (Table 2).

Hyponatremia, defined as serum Na < 136 mMol/l, was identified in 47.72% of the HF patients. Hyponatremia was present in 10% of NHYA class I patients, 25% of NYHA class II patients, 54.54% of NYHA class III patients, and 100% NYHA class IV patients.

**Figure 1:**
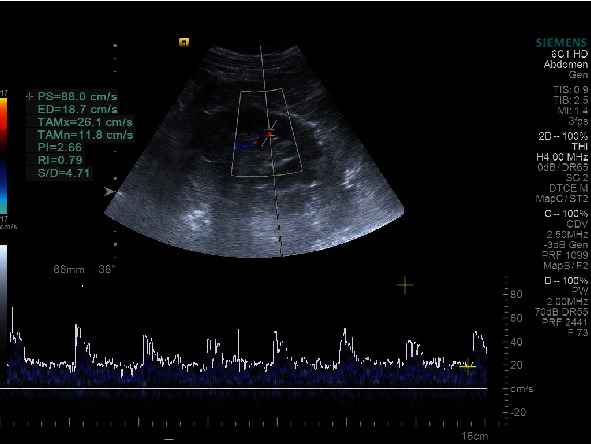
IRVF; continuous pattern.

**Figure 2:**
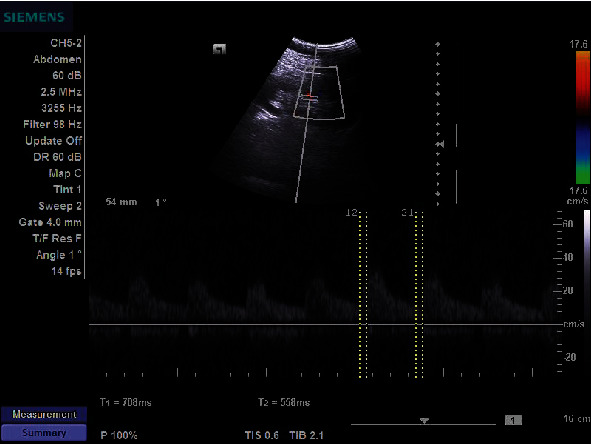
IRVF; pulsatile pattern.

**Figure 3:**
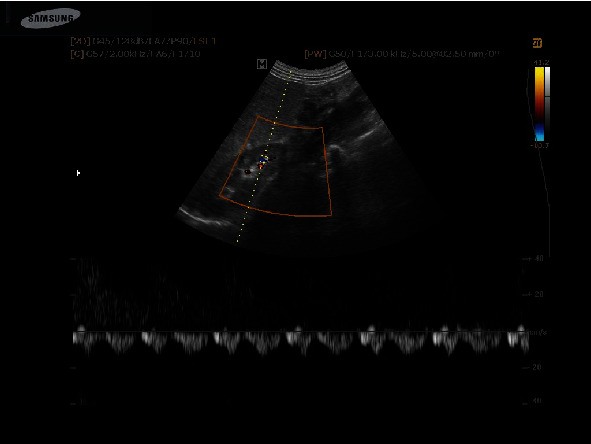
IRVF; biphasic pattern.

**Figure 4:**
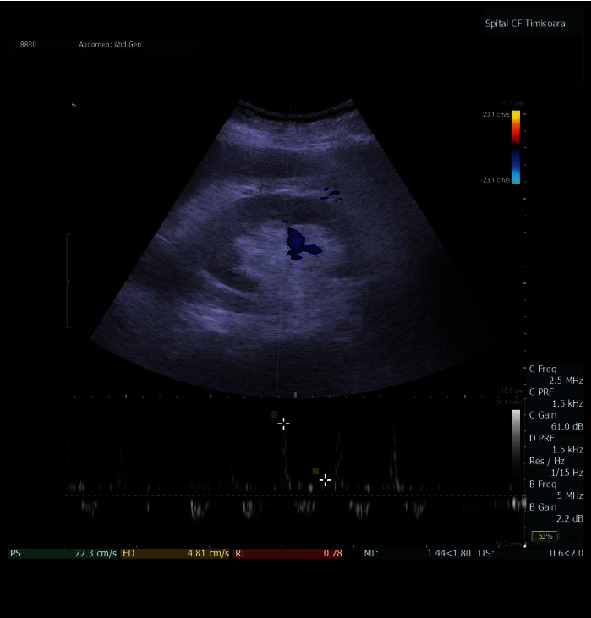
IRVF; monophasic pattern.

**Figure 5:**
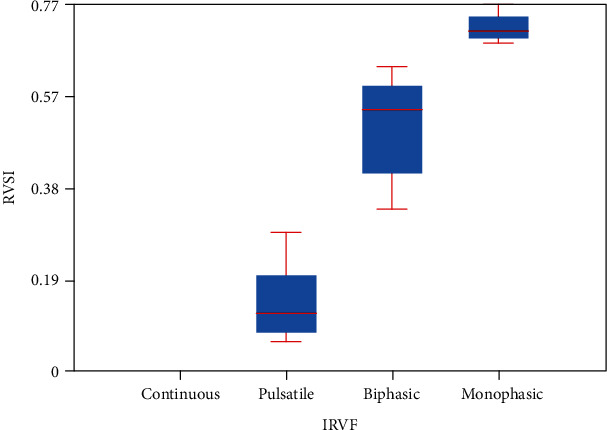
Mean values of RVSI depending on the IRVF pattern.

The study of the intrarenal arterial vascularization showed elevated RRI values in patients with HF versus controls (*p* < 0.0001). IRVF assessed by intrarenal Doppler ultrasonography showed continuous (Figure 1), pulsatile (Figure 2), biphasic (Figure 3), or monophasic (Figure 4) patterns. Healthy controls showed only a continuous pattern. Analysing the IRVF pattern, four types of renal venous flow were identified as continuous (17 patients), pulsatile (12 patients), biphasic (11 patients), and monophasic (4 patients). The mean values of RVSI in HF patients were 0 (continuous pattern of IRVF), 0.14 ± 0.07 (pulsatile pattern of IRVF), 0.51 ± 0.11 (biphasic pattern of IRVF), and 0.72 ± 0.03 (monophasic pattern of IRVF) (*p* < 0.0001) (Table 3, Figure 5).

Reduction of LVEF leads to pathophysiological changes that accompany HF, highlighting increases in NT-proBNP and serum creatinine and reduction of serum natremia values. The kidney's vascular response to these changes consists of increased intrarenal IR as well as RVSI (Table 4).

Decreased cardiac output and pulmonary and systemic congestion defined the hemodynamic profile of HF. Consecutive reduced arterial renal flow caused an increase of RRI. But RRI may be increased due to other condition, such as hypertensive nephroangiosclerosis, arteriolosclerosis, and arterial stiffness. In our HF patients, statistically analysis did not reveal significant differences in RRI values between NYHA classes (*p* = 0.736). But systemic congestion caused dilution hyponatremia and RVSI changes (*p* ≤ 0.001).

**Figure 6:**
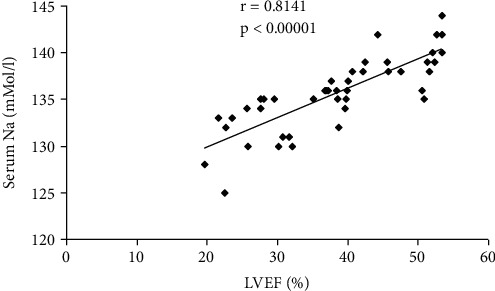
Correlations between serum Na and LVEF.

**Figure 7:**
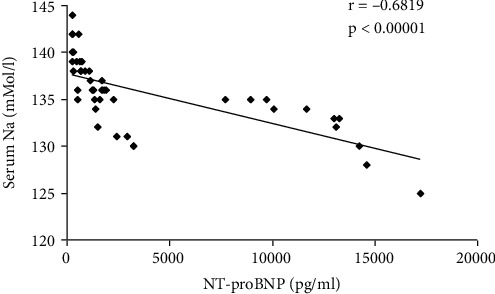
Correlations between serum Na and NT-proBNP.

**Figure 8:**
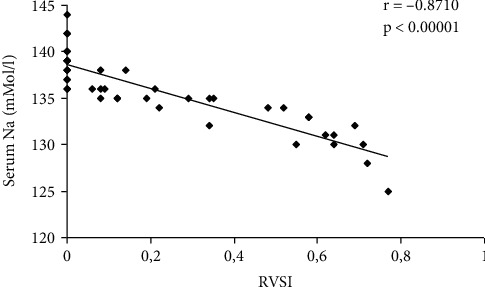
Correlations between serum Na and RVSI.

The correlations between serum Na and LVEF, NT-proBNP, serum creatinine, interlobar arteries RRI, and interlobar venous RVSI are presented in Table 5 and Figures 6–8.

Among the patients with serum Na < 135 mMol/l, 9 died during a period of 12 months. Only one patient with serum Na > 135 mMol/l died during the same period of evolution (OR 16.50; 95% CI: 1.8606-146.5237).

Renal venous congestion had a poor prognosis of these patients. Among the patients with pulsatile, biphasic, and monophasic patterns of IRVF, 9 died during the same period of evolution (OR 9; 95% CI: 1.0249, 79.03350).

## 4. Discussion

The kidneys have an important role in maintaining the hydroelectrolytic and acid-base balance, in the hemoglobin synthesis and in the metabolic waste product clearance. The kidneys interact with many organs in order to maintain homeostasis of the whole organism. One of these organs is represented by the heart. Cardiac dysfunction has repercussions on kidney function, which in turn contributes to the worsening of heart function. Ronco et al. defined cardiorenal syndrome as “a complex pathophysiological disorder of the heart and the kidneys whereby acute or chronic dysfunction in one organ may induce acute or chronic dysfunction in the other organ” [21]. Reducing the LVEF leads to decreased cardiac output, tissue hypoperfusion, and, then, the onset of neurohormonal mechanisms, which will cause sodium retention, with the occurrence of systemic congestion [6].

The present study, performed on 44 HF patients in different severity classes, showed a strong correlation between cardiac and renal dysfunction, as well as hydroelectrolytic disturbances (dilutional hyponatremia). On the other hand, hyponatremia and intrarenal venous congestion were associated with high mortality among the HF patients.

The studied HF patients presented low values of serum natremia than the patients with normal cardiac function (*p* < 0.0001). In parallel with the increase of the severity of the NYHA functional class, the reduction of the serum values of sodium was found, installing the dilutional hyponatremia (*p* ≤ 0.001). A significant correlation was identified between the serum sodium values and the LVEF (*r* = 0.8141, *p* < 0.00001).

Among the HF patients, 18-27% presented hyponatremia at the moment of hospital admission [6]. Hyponatremia is associated with increased morbidity and mortality [22].

Kiliçkiran Avci et al. reported that LVEF is lower in hyponatremic HF group of patients than in normonatremic one (*p* ≤ 0.002) [23]. In another study, published by Velat et al., it was identified that among HF patients with LVEF ≤ 45% hyponatremia was present in 48.1% of them, while normal serum natremia was present in 37.7% (*p* = 0.02) [24].

Several studies identified the relationship between hyponatremia and morbidity and mortality in HF patients. Lee and Packer, studying 203 patients with severe HF, reported that the patients with hyponatremia had a shorter survival than the patients with normal serum Na (164 days versus 373 days, *p* = 0.006) [22]. In their meta-analysis, Rusinaru et al. showed that the risk of death in HF patients increases linearly with the reduction of serum sodium values. The authors concluded that the low values of serum sodium constituted an independent predictive risk factor of death in HF with reduction ejection fraction (HR 1.69; 95% CI: 1.50-1.91) and HF with preserved ejection fraction (HR 1.40; 95% CI: 1.10-1.79) [25]. Deubner et al., analysing 1000 consecutive HF patients for a period of 5.1 years, identified that hyponatremia was associated with a significantly increased risk of mortality (HR 2.10; 95% CI: 1.60-2.77) [26]. The presence of hyponatremia in HF is associated with readmission to the hospital, increased length of hospitalization, increased rate of complications, and high costs [27]. The study performed by Yoo et al. on HF patients identified that the mean admission sodium level was 138 ± 4.7 mMol/l. About 16.8% of patients had serum natremia under 135 mMol/l. The HF patients with hyponatremia showed a higher 12-month mortality (27.9% vs. 14.6%, *p* < 0.001). The authors highlighted that the hyponatremia represented an independent predictor of 12-month mortality (HR 1.72; 95% CI: 1.12-2.65) [28]. Adrogué showed that hyponatremia represented the most common electrolyte disorder among HF patients; its frequency was associated with the severity of the functional class of HF, also representing an important factor for morbidity and hospital readmissions [29].

Mohammed et al. identified hyponatremia under 135 mMol/l in 24% of the hospitalized HF patients. All these patients presented high values of NT-proBNP than the patients with normal values of serum Na (*p* < 0.05). The authors demonstrated that hyponatremia represented an independent predictor of 1-year mortality (HR 1.72; 95% CI: 1.22-2.37; *p* < 0.001). On the other hand, high values of NT-proBNP are associated with high rates of mortality, too (HR 1.49; 95% CI: 1.10-2.00; *p* < 0.009). The association between hyponatremia and high values of NT-proBNP was correlated with the highest rates of 1-year death (*p* < 0.001) [30].

The present study showed a negative correlation between the serum natremia and NT-proBNP (*r* = −0.68198, *p* < 0.00001).

The HF patients with elevated values of natriuretic peptides (B-type natriuretic peptide and NT-proBNP) have volume overload and high filling pressure [13]. In the study performed by Velat et al., NT-proBNP levels, marker of HF severity, were significantly higher in hyponatremic than in nonhyponatremic HF patients (*p* = 0.006). Levels of NT-proBNP levels presented inverse significantly correlations with the glomerular filtration rate and LVEF [24].

Intrarenal vascularization, assessed by Doppler ultrasonography of interlobar vessels, is a marker of kidney morphologic and functional changes [31].

Intrarenal resistive index (RRI) is a measure of vascular and parenchymal kidney abnormalities [31]. This index was identified as having a prognosis role in renal parenchymal diseases and high values of RRI being registered in vascular and tubulointerstitial renal diseases [32]. In the present study, the mean value of RRI was higher in the HF group than in controls (0.71 ± 0.02 versus 0.66 ± 0.02, *p* < 0.0001), proving a negative correlation between the serum natremia and RRI (*r* = −0.44509, *p* ≤ 0.002). Ciccone et al. showed that the increased values of RRI were associated with cardiac and renal events at univariate (HR 1.14; 95% CI: 1.09-1.19; *p* < 0.001) as well as at multivariate Cox regression analysis (HR 1.08; 95% CI: 1.02-1.13; *p* = 0.004) [31]. In HF patients, RRI above 0.75 is associated with unfavorable prognosis, both cardiac and renal [32]. Only one of the studied patients had RRI over 0.75, and within the 365-day follow-up period, he died. RRI did not show statistically significant differences between NYHA functional classes of HF (*p* = 0.736), because the value of RRI was largely influenced by the disease that generates HF (arterial hypertension, atherosclerosis) [7].

The analysis of intrarenal Doppler venous flow (IRVF) patterns assessed the intrarenal congestion in HF patients and brought additional information to the exploration of arterial vascularization. The intrarenal venous congestion in HF patients has only been studied for a few years. Husain-Syed et al. identified the role of kidney venous congestion in worsening of the renal function in HF patients and proposed that an adequate control of congestion is an important goal in HF therapy [7]. Under physiological conditions, the IRVF has a continuous pattern. Systemic congestion and increased in central venous pressure cause a discontinuous IRVF (pulsatile, biphasic, and monophasic), depending the right atrial pressure. Discontinuous patterns of IRVF venous flow imply an unfavorable prognosis [32].

Renal venous stasis index (RVSI) is a new ultrasonographic parameter, which allows appreciation the proportion of the cardiac cycle during which there is no renal venous flow. It is calculated based on the following formula: RVSI = (cardiac cycle time [msec] − venous flow time [msec])/cardiac cycle time [msec] [7].

In this study, continuous pattern of IRVF was identified in all controls and in 17 HF patients, whereas discontinuous pattern in 27 HF patients (12 cases with pulsatile pattern, 11 cases with biphasic pattern, and 4 patients with monophasic pattern). IRVF had prognostic value, because among the HF patients with pulsatile, biphasic, and monophasic patterns of IRVF, 9 died during the 365 days (OR 9; 95% CI [1.0249, 79.0335]). Wilson Tang and Kitai showed in their study that the HF patients with continuous intrarenal venous pattern had favourable prognosis, having a 12-month survival of over 95%. But the HF patients with discontinuous pattern of IRVF had a poorer prognosis, with survival at 12 months less than 40% [33]. IRVF pattern represents a prognosis predictor in HF patients; it was correlated with the serum natremia (*p* < 0.05) and logBNP (*p* = 0.009) [34]. Puzzovivo et al. demonstrated that the discontinuous pattern of IRVF has a negative prognostic in HF patients (*p* < 0.001) [35]. In another study, performed by Trpkov et al., discontinuous IRVF was associated with systemic congestion in HF patients and high values of serum creatinine [36]. In our study, it showed that RVSI increased with the severity of the NYHA functional class (*p* ≤ 0.001), correlating with the serum natremia (*r* = −0.8710, *p* < 0.00001). But RVSI increased statistically significantly with the type of IRVF, in ascending order, as follows: continuous, pulsatile, biphasic, and monophasic (*p* < 0.0001). The same result was reported by Husain-Syed et al. in their study [7].

The present study has some limits. First, the relatively small number of HF patients represents one of its limits, because, at the time the study began, no other team of researchers in Romania researched this topic. On the other hand, the patients with decompensated HF and acute or chronic kidney diseases were not included in this study.

## 5. Conclusion

The patients with heart failure that presented dilutional hyponatremia correlated with cardiac dysfunction (highlighted by left ventricular ejection fraction reduction and NT-proBNP increase) and, on the other hand, with intrarenal venous congestion. Hyponatremia and renal venous congestion represent negative prognostic factors in HF patients.

## Figures and Tables

**Table 1:** Demographic data in pSS patients and controls.

Parameter	Value (mean ± standard deviation)	
	pSS patients	Controls
Sex (*n* (%))	44	44
Males	24 (54.54%)	24 (54.54%)
Males	20 (45.45%)	20 (45.45%)
Mean age (years)	63.52 ± 7.03	60.38 ± 7.46
Etiology of HF	(i) Ischaemic heart disease (including previous myocardial infarction) (18 patients) (ii) Arterial hypertension (17 patients) (iii) Primary dilated cardiomyopathy (5 patients) (iii) Rheumatic heart disease (2 patients) (iv) Degenerative valvular disease (2 patients)	—
The drugs used by the HF patients in the moment of investigation	(i) Angiotensin-converting enzyme inhibitors (19 patients) (ii) Angiotensin receptor blockers (25 patients) (iii) Beta-blockers (37 patients) (iv) Diuretics (44 patients) (v) Mineralocorticoid receptor antagonist (26 patients)	—

**Table 2:** Parameters assessed in HF patients and controls.

Parameter	HF patients	Controls	*p*
LVEF (%)	38.35 ± 10.22	59.80 ± 4.31	<0.0001
NT-proBNP (pg/ml)	3929.11 ± 5044.27	183.38 ± 54.34	<0.0001
Serum Na (mMol/l)	135.63 ± 3.94	140.52 ± 2.12	<0.0001
Serum creatinine (mg/dl)	1.36 ± 0.46	0.97 ± 0.12	<0.0001
eGFR (ml/min/1.73m^2^)	52.09 ± 17.68	68.38 ± 29.39	<0.001

**Table 3:** Intrarenal vascular parameters in HF patients and controls.

Parameter	HF patients	Controls	*p*
IR	0.71 ± 0.02	0.66 ± 0.02	<0.0001
IRVF pattern			
(i) Continuous	17 patients	44 patients	
(ii) Pulsatile	12 patients		
(iii) Biphasic	11 patients		
(iv) Monophasic	4 patients		
RVSI	0.23 ± 0.26	0	<0.0001
(i) Continuous pattern	0		
(ii) Pulsatile pattern	0.14 ± 0.07		
(iii) Biphasic pattern	0.51 ± 0.11		
(iv) Monophasic pattern	0.72 ± 0.03		
	*p* < 0.0001		

**Table 4:** Monitored parameters in NYHA functional classes.

Parameter	HF patients				*p*
	NYHA I	NYHA II	NYHA III	NYHA IV	
LVEF (%)	52.16 ± 1.04	42.2 ± 2.93	35.01 ± 3.23	24.92 ± 3.15	≤0.001
NT-proBNP (pg/ml)	338.10 ± 104.52	987.67 ± 341.22	2185.83 ± 695.21	12145.8 ± 2811.55	≤0.001
Serum Na (mMol/l)	139.5 ± 2.75	137.16 ± 2.62	133.90 ± 2.77	132.18 ± 3.25	≤0.001
Serum creatinine (mg/dl)	0.968 ± 0.11	1.16 ± 0.09	1.26 ± 0.11	2.05 ± 0.41	≤0.001
eGFR (ml/min/1.73 m^2^)	74.2 ± 10.76	54.91 ± 10.25	50.63 ± 4.98	30.36 ± 8.98	≤0.001
IR	0.69 ± 0.01	0.71 ± 0.01	0.70 ± 0.01	0.73 ± 0.16	0.736
RVSI	0.012 ± 0.03	0.09 ± 0.11	0.26 ± 0.28	0.54 ± 0.16	≤0.001

**Table 5:** Correlations between serum Na and monitored parameters.

Correlation between serum Na and	*r*	*p*
Intrarenal RVSI	-0.87104	<0.00001
Intrarenal RI	-0.44509	≤0.002
Serum creatinine	-0.68983	<0.00001
NT-proBNP	-0.68198	<0.00001
LVEF	0.8141	<0.00001

## Data Availability

All the processed data were extracted from the records of hospitalized patients.
